# Algorithm for early diagnosis of viral hepatitis C

**DOI:** 10.1093/eurpub/ckac131.557

**Published:** 2022-10-25

**Authors:** I Pakov, K Terzieva, D Shalamanov, M Karcheva, G Gancheva

**Affiliations:** 1 Department of Infectious Diseases, Epidemiology, Parasitology and Tropical Medicine, Medical University-Pleven, Pleven, Bulgaria; 2 Scientific Centre of Military Epidemiology and Hygiene, Military Medical Academy, Sofia, Bulgaria

## Abstract

**Issue:**

Viral hepatitis C (VH C) is a global problem of the public health. Different interventions for achieving the Global Health Sector Strategy on viral hepatitis targets (65% reduction in HCV-related deaths, 90% in new infections and 90% of infections diagnosed by 2030) were considered. Increased diagnosis and treatment rates would be required to achieve these targets in all countries, even with the introduction of high sustained viral response therapies.

**Description of the problem:**

The latest global HCV disease burden estimates showed that about 71.1 million people worldwide are viremic, corresponding to a prevalence of 1%. The prevalence of HCV is not homogenous: the WHO Eastern-Mediterranean Region is with the highest number of infected subjects (15 millions), followed by the European Region (14 million). In Bulgaria, the incidence of VH C is 0.63-1.30 at 100 000 population (2008-2020) and in Pleven region is 0.38-3.8, respectively.

**Results:**

Retrospective study was conducted upon epidemiological, demographic, clinical, laboratory and viral characteristics in fifty cases of VH C confirmed with positive anti-HCV, evaluated by ELISA. Thirty eight of cases were hospitalized in different clinics of the University Hospital “Dr Georgi Stranski”-Pleven (2017-2018) and remainders were blood-donors registered in Regional Center of Transfusion Hematology-Pleven. Surgical interventions (26.32%), blood infusions (23.68%) and hemodialysis (15.79%) were at highest risk for VH C. Twenty five hospital patients were with chronic VH C (66%), five with cirrhosis (13%) and eight (21%) with acute hepatitis C. Twenty of the patients (53%) were asymptomatic about hepatitis and were hospitalized because of different comorbidities (p < 0.0005).

**Lessons:**

We propose an algorithm for early diagnosis of VH C based on mandatory screening for anti-HCV in risk groups, especially before invasive procedures. The early detection of HCV infection will reduce the complications and nosocomial infections.

**Key messages:**

• Different scenarios developed to achieve the WHO Targets in all countries assume an implementation of national policies to prevent new infections and to diagnose current infections through screening.

• The early detection of HCV infection will reduce the complications and nosocomial infections.

